# Renipuncture in Albiminuria

**Published:** 1897-04

**Authors:** 


### RENIPUNCTURE IN ALBUMINURIA.

The safety with which exploratory incisions can be performed and the freedom from danger involved in antiseptic surgery have brought some diseases formerly considered strictly medical under the surgeon's charge.

Reginald Harrison relates in the MEDICAL RECORD several cases in which an operation for supposed renal calculi had resulted in the disappearance of an existing albuminuria, and he justly inferred that operative procedures might help some forms of albuminuria. In these cases he found the capsule of the kidney tense, like the surface of a ripe plum.

Accordingly, he made several incisions into the kidney and thus reduced the tension, with the satisfaction of seeing the wound gradually heal with no return of the albuminuria. His cases were all albuminurics but they were not operated on for the relief of that trouble, but for some other diseased condition.

That this form of treatment is right, analogy will show. The eye is a delicate and sensitive organ which may become glaucomatous from intra-ocular tension; the testicle not infrequently becomes inflamed and tense, thus involving the functioning of that organ. In both these organs incision to reduce the tension is the proper treatment which gives the best hopes for a cure. The structural arrangements of the kidney are such that an incision for increased tension would be the most natural operation and it is indicated in a large number of cases.—*Maryland Medical Journal.*

